# Exploring the Role of Long Non-Coding RNAs in Mediating Cisplatin Resistance in Glioma/Glioblastoma Cells

**DOI:** 10.3390/ijms27136010

**Published:** 2026-07-04

**Authors:** Hadi Sahrai, Reza Mosaddeghi-Heris, Nasrin Forghani, Ali Norouzi, Sahand Zare, Hamed Aghazadeh, Kimia Bagheri, Rebecca Kocsis, Firoz Ahmed, Niloofar Taheri, Shahab Uddin, Maryam Farzaneh

**Affiliations:** 1 Research Center for Evidence-Based Medicine, Iranian EBM Centre: A JBI Centre of Excellence, Faculty of Medicine, Tabriz University of Medical Sciences, Tabriz 5166614711, Iran; hsahrai1997@gmail.com; 2 Neurosciences Research Center (NSRC), Tabriz University of Medical Sciences, Tabriz 5166614711, Iran; rezamosaddeghi1375@gmail.com (R.M.-H.); nasrin.forghani98@gmail.com (N.F.); 3 Student Research Committee, Tabriz University of Medical Sciences, Tabriz 5166614711, Iran; alii.norouzi97@gmail.com; 4 Clinical Research Development Unit of Tabriz Valiasr Hospital, Tabriz 5166614711, Iran; sahandzare@gmail.com; 5 Faculty of Pharmacy, Tehran University of Medical Sciences, Tehran 1417614411, Iran; hamed.aghazadeh@ut.ac.ir; 6 Faculty of Pharmacy, Tabriz University of Medical Sciences, Tabriz 5166614711, Iran; 7 Infectious Diseases and Tropical Medicine Research Center, Health Research Institute, Babol University of Medical Sciences, Babol 4717647745, Iran; bagheriikimia@gmail.com; 8 Department of Psychiatry and Behavioral Sciences, Stanford University, Palo Alto, CA 94305, USA; rkocsis@stanford.edu; 9 Translational Research Institute, Academic Health System, Hamad Medical Corporation, Doha P.O. Box 3050, Qatar; fahmed25@hamad.qa (F.A.); skhan34@hamad.qa (S.U.); 10 Fertility, Infertility and Perinatology Research Center, Ahvaz Jundishapur University of Medical Sciences, Ahvaz 6135733118, Iran; 11 Clinical Research Development Unit, Imam Khomeini Hospital, Ahvaz Jundishapur University of Medical Sciences, Ahvaz 6135733118, Iran

**Keywords:** gliomas, lncRNAs, cisplatin, resistance, apoptosis

## Abstract

Malignant gliomas are highly aggressive primary brain tumors for which the therapeutic efficacy of cisplatin is frequently limited by intrinsic or acquired drug resistance. Despite advances in adjuvant therapies, overcoming chemoresistance remains a major challenge in the treatment of these malignancies. Emerging evidence indicates that long non-coding RNAs (lncRNAs), a class of non-protein-coding transcripts involved in gene regulation, play important roles in modulating treatment responses. Several lncRNAs, including differentiation antagonizing non-protein-coding RNA (DANCR), HOXD antisense growth-associated long non-coding RNA (HOXD-AS1), MEG3, MALAT1, and HOTAIR, have been implicated in pathways associated with glioma progression and therapeutic resistance. In particular, DANCR has been reported to promote cisplatin resistance in glioma cells through suppression of apoptosis and activation of pro-survival signaling pathways. This review summarizes current evidence regarding the roles of lncRNAs in cisplatin resistance, highlighting mechanisms such as regulation of drug transport, DNA damage repair, apoptosis, cancer stem-cell maintenance, and signaling pathways associated with treatment adaptation. We also discuss current limitations, challenges for clinical translation, and gaps in the existing evidence. A better understanding of lncRNA-mediated resistance mechanisms may facilitate the identification of novel therapeutic targets and inform future studies aimed at overcoming cisplatin resistance in malignant gliomas.

## 1. Introduction

Gliomas are the most prevalent malignant brain tumors, with glioblastoma (GBM) accounting for the majority—about 57.7% of all gliomas. GBM represents approximately 48.6% of central nervous system tumors, and despite ongoing advances in diagnosis and treatment, its management remains a significant challenge for clinicians [1,2]. Gliomas are classified into grades I to IV, with low-grade gliomas exhibiting slower growth and more favorable outcomes. In contrast, GBM is a tumor characterized by abnormal growth, a strong tendency to develop therapy resistance, and early recurrence. The clinical manifestations of both glioma and GBM include headaches, seizures, and neurological deficits; however, these symptoms typically appear at advanced stages [3,4]. The incidence rate of GBM is higher in males than in females and is greater in developed and Western countries compared to developing countries. Overall, the incidence rate of GBM is less than 10 per 100,000 individuals [5]. The common risk factors include exposure to ionizing radiation, certain pesticides and agricultural chemicals such as organochlorides and alkylureas combined with copper sulfates, and prior treatment for acute lymphoid leukemia (ALL) [6,7,8]. Genetic predisposition has been reported in only 5–10% of cases [9].

Macroscopically, GBM is highly heterogeneous, featuring multifocal hemorrhage, necrosis, and cystic or gelatinous areas [10]. Ongoing and recent investigations have identified molecular correlates underlying these clinical characteristics. Hallmark alterations of primary GBM include epidermal growth factor receptor (EGFR) gene mutation and amplification; overexpression of mouse double minute 2 (MDM2); deletion of p16; loss of heterozygosity (LOH) of chromosome 10q harboring phosphatase and tensin homolog (PTEN); and TERT promoter mutations. According to the 2021 WHO Classification of Tumors of the Central Nervous System, glioblastoma is defined as an IDH-wild-type diffuse astrocytic glioma with characteristic molecular features such as TERT promoter mutation, EGFR amplification, or combined chromosome 7 gain/chromosome 10 loss. Tumors previously termed “secondary glioblastomas” are now classified as astrocytoma, IDH-mutant, CNS WHO grade IV [11].

Recent advances in imaging techniques, particularly magnetic resonance imaging (MRI), have significantly improved the evaluation of hemodynamic changes, tissue architecture, and cellular metabolism in gliomas. Nuclear medicine techniques, such as single-photon emission computed tomography (SPECT) and positron emission tomography (PET), are also used as efficient tools for distinguishing active tumors from therapy-related changes [12]. Imaging biomarkers, derived from computed tomography (CT), MRI, or PET, provide quantitative parameters that objectively describe biological processes, pathological changes, and treatment responses in different situations [13].

Treatment of GBM remains challenging and typically includes surgical resection, chemotherapy, radiotherapy, and symptomatic management of seizures and headaches [14]. Despite aggressive multimodal therapy, the median survival rate is 14–16 months, with approximately 50% of patients surviving beyond 15 months after initial diagnosis. Furthermore, patients with GBM demonstrate relapse after 7 months of starting treatment [15,16]. The current standard of care for GBM involves maximal safe surgical resection followed by concurrent temozolomide (TMZ; 75 mg/m^2^/day for 6 weeks) and radiotherapy (RT; 60 Gy in 30 fractions), followed by six maintenance cycles of TMZ (150–200 mg/m^2^/day for the first 5 days of a 28-day cycle—sdTMZ) [17]. Although Li et al. have shown promising preclinical/immunomodulatory effects of cisplatin, it is not clinically used as a first-line treatment for GBM [18].

Ongoing studies have revealed that dysregulated expression of long non-coding RNAs (lncRNAs) plays an essential role in cisplatin resistance [19]. lncRNAs, which are >200 nucleotides (nt) long and lack a significant open reading frame, regulate various biological pathways and cellular processes at the epigenetic, transcriptional, and post-transcriptional levels [20]. Cisplatin resistance may arise when cancer cells accumulate less of the drug internally, either by limiting its entry or by enhancing its removal. For example, decreased expression of the copper transporter protein CTR1 is associated with reduced cisplatin uptake, thereby contributing to drug resistance. Conversely, cisplatin export from cells is facilitated by P-type ATPases such as ATP7A and ATP7B, as well as multidrug resistance–associated proteins (MRPs) in the cell membrane. Increased activity or expression of these efflux transporters is considered one of the key mechanisms underlying cisplatin resistance [21].

Cisplatin scavenging by intracellular detoxification is another major mechanism of cisplatin resistance, in which glutathione (GSH) plays an important role. Overexpression of enzymes involved in GSH synthesis and GSH conjugation has been associated with cisplatin resistance. Moreover, activation of DNA damage repair systems, such as the nucleotide excision repair system, can attenuate the apoptotic process, leading to cisplatin resistance. Increased expression of nucleotide excision repair proteins, including the XPF–ERCC1 complex, has been reported to reduce the efficacy of platinum-based therapy [22,23]. Studies have also indicated that autophagy contributes to cisplatin resistance, and downregulation of RB suppresses cisplatin-induced autophagy [24]. Previous studies have reported that DANCR is highly expressed in glioma tissues and cells and plays an important role in glioma progression and cisplatin resistance [25]. In recent years, numerous studies have proposed that lncRNAs are involved in embryonic development and in the etiology of various human diseases, including cancer [26]. Advanced sequencing technologies have revealed that numerous lncRNAs are dysregulated or aberrantly expressed in multiple types of cancers. These lncRNAs act as key factors in cancer development and progression by regulating cell proliferation, apoptosis, metastasis, and angiogenesis [27]. Given their critical role in tumor cell survival and death, it is plausible that lncRNAs also modulate cell sensitivity to chemotherapy, which targets tumor cells by inhibiting cell growth and inducing apoptosis. For example, lncRNAs have been reported to contribute to doxorubicin resistance through regulating MDR1 expression [28]. Considering that various molecular networks are dysregulated during the development of drug resistance in GBM, and that lncRNAs serve as key regulators of tumor signaling pathways, investigating their role in overcoming GBM resistance is crucial. The delicate and infiltrative nature of GBM further limits the effectiveness of surgical resection, as cancer cells often invade surrounding tissues before complete removal is feasible. Hence, new therapeutic strategies are urgently needed. Considering the multifaceted role of lncRNAs in modulating cancer progression, treatment response, and the biological behavior of GBM, targeting lncRNAs represents a promising approach. Both pharmacological and gene-based methods can be employed to regulate lncRNA activity. In this review, we discuss the significance of lncRNAs in GBM, focusing on how their dysregulation influences various downstream signaling pathways, targets, tumor invasion, and cisplatin resistance.

## 2. The Literature Search Strategy

A narrative literature review was conducted using PubMed, Scopus, and Web of Science up to January 2026. Search terms included “glioblastoma”, “glioma”, “cisplatin resistance”, “long non-coding RNA”, “lncRNA”, “DANCR”, “chemoresistance”, and related combinations. Studies investigating lncRNAs in glioma biology, cisplatin resistance, DNA repair, apoptosis, drug transport, stemness, and associated signaling pathways were considered. We only included studies written in English.

## 3. Detail Molecular Mechanisms of Cisplatin Resistance in GBM

Cisplatin exerts cell-cycle non-specific cytotoxicity by forming covalent platinum–DNA adducts, primarily with the purine bases guanine and adenine. Platinum-based drugs, particularly cisplatin, are used to treat a wide range of malignancies [29]. Despite consistent initial response rates, cisplatin treatment often results in the development of chemotherapy resistance, leading to treatment failure. DNA is the primary intracellular target of cisplatin, and restriction of cisplatin–DNA binding contributes to drug resistance. After nuclear entry, cisplatin can be inactivated by metallothionein through binding to its metal core intermediate, thereby reducing its cytotoxic activity. In addition to classical mechanisms, such as increased drug clearance, enhanced DNA damage repair, and alterations in cell cycle and apoptotic pathways, other cellular adaptations have been implicated. These include increased clearance of damaged proteins, enhanced protein glycosylation, increased glycolytic processes, and dysregulation of oxidative phosphorylation, all of which may contribute to cisplatin resistance in GBM [30].

### 3.1. Molecular Mechanisms of Cisplatin Cytotoxicity

#### DNA Adduct Formation, Cell-Cycle Arrest, and Apoptosis

Cisplatin induces covalent DNA adducts that activate the DNA damage response (DDR). This process involves activation of the ATM and ATR pathways, leading to cell-cycle arrest, DNA repair, or cell death when the damage is severe. p53 dysfunction in GBM commonly results from mutations within the DNA-binding domain (frequently exons 5–8) and may also be associated with loss of chromosome 17p. p53 can be regulated by stress-responsive MAPK pathways, particularly JNK and p38, while ERK signaling may contribute in a context-dependent manner. As a result, loss of p53 leads to genomic instability. Taken together, cisplatin-induced cancer cell death is mediated through a series of biochemical effects and molecular mechanisms that include DNA adduct formation, p53 activation, MAPK signaling, and cell-cycle arrest. Cisplatin also promotes cell-cycle arrest and apoptosis by modulating the expression of apoptosis-related proteins, including downregulation of the anti-apoptotic protein BCL2 and activation of mitochondrial apoptotic pathways [31].

### 3.2. Hallmarks of Chemoresistance in GBM

#### 3.2.1. Enhanced Drug Efflux and Reduced Accumulation

ABC transporters consist of seven subfamilies (ABC-A to -G) that encompass 49 proteins and play multiple biological roles. For example, ABCB1 restricts the penetration of numerous chemotherapeutic agents into the brain by actively transporting substrates from BBB endothelial cells back into the circulation. However, the broad substrate specificity of ABC transporters remains a major obstacle to effective drug delivery and therapeutic efficacy in GBM. Nevertheless, they could be potential targets that warrant further evaluation regarding alternative strategies to enhance the efficacy of platinum-based and other chemotherapeutic agents [32]. Cisplatin is limited by its reduced ability to cross the blood–brain barrier (BBB), intrinsic/acquired drug resistance, development of multidrug resistance (MDR) mechanisms by cancer cells, and low bioavailability [33]. The blood–brain barrier has always been the first barrier to GBM treatment, providing a physical and biological barrier that reduces brain exposure to drugs [34]. In addition to the structural barrier provided by endothelial tight junctions, ABC transporters at the BBB constitute a critical biochemical barrier to drug delivery [35]. These include ABCB1 (glycoprotein P, MDR1), ABCC1 (MRP1—multidrug resistance protein 1), ABCC2 (MRP2), ABCC4 (MRP4), ABCC5 (MRP5), and ABCG2 (BCRP—breast cancer resistance protein) in cisplatin-resistant cells [36]. Only three of these, ABCC2, ABCC5, and ABCC6, have been reported to contribute directly to cisplatin transport and resistance. Upregulation of ABC transporters in cisplatin-resistant cells may contribute to cross-resistance against multiple chemotherapeutic agents [37].

#### 3.2.2. Enhanced DNA Repair Through Nucleotide Excision Repair

Nucleotide excision repair (NER) is a versatile and essential DNA repair mechanism that repairs a diverse range of DNA damage that causes helix deformation. NER is particularly well-known for repairing UV-induced DNA damage [38]. Cisplatin reacts with DNA bases to produce various forms of cisplatin–DNA adducts [39]. NER is involved in the recognition and removal of cisplatin-induced intrastrand DNA adducts, whereas repair of interstrand crosslinks requires coordinated activity of the Fanconi anemia, homologous recombination, and NER pathways [40]. Elevated expression of the ERCC1-XPF endonuclease complex has been associated with reduced cisplatin sensitivity in cancer models, suggesting that efficient excision of cisplatin-induced DNA lesions may contribute to chemoresistance [41]. Despite its clinical utility, the efficacy of cisplatin is frequently limited by dose-limiting toxicity and the development of acquired resistance [42]. For this reason, targeting DNA repair in GBM has attracted new attention [43]. However, the tumor-suppressing effect of PARP inhibitors is low in IDH1/2-wild-type gliomas. In IDH-wild-type gliomas, intact BRCA1/2-mediated homologous recombination enables efficient repair of DNA double-strand breaks, thereby reducing susceptibility to PARP inhibition [44].

#### 3.2.3. Evasion of Apoptosis: Explaining BCL2 Dysregulation

Cisplatin binds DNA in several ways and disrupts cell division through mitosis. Damaged DNA activates DNA repair mechanisms, which in turn activate apoptosis when repair is impossible [45]. Dysregulated p53 pathway components have been linked to several processes in GBM, such as cell invasion, proliferation, evasion of apoptosis, or cell stemness [46]. Evasion of treatment-induced apoptosis is a hallmark of acquired resistance. Inhibition of apoptosis is a crucial step in the transition of normal cells to malignant cells. Among the key regulators of apoptosis, the B-cell lymphoma 2 (BCL2) protein family plays an important role. BCL2 is an anti-apoptotic protein. Overexpression of anti-apoptotic BCL2 family proteins has been reported in malignancies and is associated with reduced susceptibility to chemotherapy-induced apoptosis [45]. Significant research suggests that targeting BCL2 family members has synergistic activity with standard therapies that use cytotoxic agents, such as cisplatin, to kill cancer cells [47].

#### 3.2.4. Epithelial–Mesenchymal Transition (EMT) and Invasion

Over the past decade, researchers have successfully isolated glioblastoma stem cells (GSCs) from GBM and discovered their critical role in tumor development, maintenance, and recurrence. As a result, GSCs have become a critical target for novel therapies due to their ability to resist standard therapies and contribute to relapse of the malignancy [48]. EMT-inducing transcription factors belong to three distinct protein families: the SNAIL family (SNAIL and Slug), the ZEB family (ZEB1 and ZEB2), and the basic helix–loop–helix (bHLH) family (TWIST1, TWIST2, and TCF3). These transcriptional regulators control the expression of EMT-related genes by activating or inhibiting their promoters. Although glioblastoma is not an epithelial malignancy, activation of EMT-like or mesenchymal transition programs has been implicated in tumor progression, invasion, stemness, and therapeutic resistance. In GBM, EMT has been implicated in tumor initiation, plasticity, and treatment resistance, making it a key factor in the pathophysiology of the disease. The process of EMT can promote tumor cell migration and invasion, facilitating the spread of cancer cells within the brain. Additionally, EMT is believed to contribute to the maintenance of cancer stem cells, which are thought to be responsible for tumor recurrence and resistance to conventional therapies [49].

#### 3.2.5. The Role of the Tumor Microenvironment and Cancer Stem Cells

A growing body of research has shown that tumor stem-cell subpopulations can develop resistance to therapies and cause malignant relapse due to their capabilities of differentiation, self-renewal, growth, and progression [50]. The mechanisms of resistance of GSCs include their quiescence, higher mitochondrial reserve, extensive DNA repair capabilities, and localization in hypoxic niches [51]. At the same time, several studies have shown that the tumor microenvironment (TME) and its interaction with GBM stem cells, through the release of extracellular vesicles, play an important role in the pathogenesis and proliferative characteristics of GBM. The TME has been proposed as a promising therapeutic target [52,53]. Within tumors, hypoxic regions arise due to rapid cell proliferation and insufficient vascular supply, compelling cancer cells to adapt through the activation of HIF-1α. This adaptation promotes angiogenesis by upregulating vascular endothelial growth factor (VEGF), a critical driver of aberrant vasculature in tumors [54,55]. Additionally, HIF-1α facilitates metabolic reprogramming [56]. Hypoxia also contributes to immune evasion by inducing programmed death-ligand 1 (PD-L1) expression, thereby suppressing anti-tumor T-cell responses [57].

## 4. Master Regulators of Cellular Processes in Cancer

lncRNAs are transcripts longer than 200 nucleotides that do not encode proteins but carry out regulatory functions. They are transcribed by RNA polymerase I (Pol I), Pol II, and Pol III and RNAs from processed introns, with typical mRNA-like processing [58,59]. In glioma, as the most common malignant carcinoma in the nervous system, dysregulated lncRNAs profoundly influence disease. lncRNAs have been shown to promote proliferation, invasion, angiogenesis, and therapy resistance [58,60]. lncRNAs act as molecular hubs (binding DNA, RNA, or proteins), and they can function as oncogenes or tumor suppressors. Aberrant lncRNA expression in glioma has been linked to altered immune signaling and poor prognosis, making them candidate biomarkers and therapeutic targets [58].

### 4.1. Biogenesis and Functional Classification of lncRNAs

Like mRNAs, many lncRNAs are co-transcriptionally 5′ capped, spliced, and 3′ polyadenylated [61]. Based on their genomic location relative to neighboring protein-coding genes, lncRNAs are commonly classified as sense, antisense, bidirectional, intronic, or intergenic (lincRNAs). Antisense lncRNAs originate from the opposite strand of protein-coding genes, whereas sense lncRNAs overlap coding loci on the same strand. Bidirectional lncRNAs are transcribed in the opposite orientation from promoters located near neighboring genes, while intronic and intergenic lncRNAs arise from intronic regions and intergenic genomic intervals, respectively [62,63,64].

lncRNAs have garnered substantial attention in cancer research. Although they exceed 200 nucleotides in length and lack protein-coding capacity, they exhibit regulatory functions analogous to those of protein-coding genes [65]. Typically transcribed by RNA polymerase II, lncRNAs possess a 5′ cap and a 3′ polyadenylated tail, and the majority contain more than two exons [66]. Despite recent advances in elucidating their regulatory roles in GBM, the full scope of their biogenesis and pathogenic mechanisms remains insufficiently characterized due to their remarkable diversity and the complexity of their molecular actions [65]. In addition, enhancer RNAs (eRNAs), a subclass of non-coding RNAs transcribed from active enhancer regions, have been shown to facilitate gene transcription by recruiting transcriptional machinery and promoting enhancer–promoter interactions [61]. However, the experimental tools themselves create problems. lncRNA loci commonly overlap or lie near other genes, so direct editing is risky. Small frameshift edits often do nothing because the transcript does not encode protein, forcing groups to remove promoters or long loci, a strategy that easily disturbs neighboring elements. CRISPR methods, therefore, bring collateral hits—overlapping architectures, intronic promoters, and bidirectional regions allow guides to cut unintended targets. Meanwhile, RNAi and antisense technologies that are effective for mRNAs perform poorly here. RISC sits mostly in the cytosol, though many lncRNAs function in the nucleus; abundant transcripts resist partial depletion; silencing tends to fade rapidly [67]. Delivery problems amplify all of these issues in vivo, and immune activation is another major concern. ASOs and siRNAs can provoke inflammatory signaling, and moving nucleic acids across the brain’s immune-protected environment is technically difficult [68]. Transient silencing, incomplete delivery, and off-target activity cloud interpretation of mechanistic findings.

### 4.2. Modes of Action: Chromatin Remodeling and Transcriptional and Post-Transcriptional Regulation

In glioma, lncRNAs have been reported to enhance gene expression epigenetically by recruiting chromatin-modifying complexes, such as EZH2/PRC2 and WD repeat domain 5/trithorax proteins, to specific loci where they function as scaffolds to regulate histone H3 trimethylation or acetylation. Through these mechanisms, lncRNAs influence key glioma traits, including tumorigenicity, proliferation, invasion, and chemoresistance [69,70].

NEAT1, predominantly nuclear, produces two isoforms—NEAT1 (3.7 kb) and NEAT1_2 (23 kb)—and promotes multiple cancers, including colorectal, breast, liver cancer, and glioma. Mechanistic studies show that NEAT1 is activated by EGFR signaling and serves as a scaffold for EZH2 recruitment [71].

In glioma models, NEAT1 has been reported to recruit EZH2 to the promoters of Axin2 and GSK3β, resulting in increased H3K27 trimethylation and activation of Wnt/β-catenin signaling [70,72]. Another lncRNA, ZFAT-AS1, derived from an imprinted locus, interacts with EZH2 to catalyze H3K27 methylation and suppress CDX2 transcription, thereby enhancing glioma cell proliferation, migration, and invasion [73]. MALAT1, a well-characterized nuclear-retained lncRNA, is predominantly recruited to nuclear speckles, where it regulates pre-mRNA splicing. Comparative analyses of U87 and U251 glioma cell lines with normal human astrocytes have shown markedly elevated MALAT1 expression in glioma cells, supporting its role as an oncogenic lncRNA in this tumor type [4,74]. Functionally, MALAT1 enhances the expression of sex-determining region Y-box 2 (SOX2), thereby promoting the viability and proliferation of glioblastoma stem cells. In addition, MALAT1 has been reported to promote glioma cell survival and inhibit apoptosis through miR-101-dependent signaling pathways [4].

### 4.3. The Oncogenic and Tumor Suppressor Paradigm of lncRNAs in Glioma

Zhang et al. [75] implicated HOTAIR in glioma pathogenesis. Noting its predominant expression in classical and mesenchymal subtypes compared with gliomas, they suggested that HOTAIR may function as a useful biomarker for distinguishing molecular subtypes based on gene-expression profiles. Analyses of HOTAIR expression, methylation, and copy-number alterations in human gliomas, including IDH-wild-type glioblastoma, indicate a positive correlation with increasing tumor grade. Notably, HOTAIR has emerged as a candidate prognostic biomarker, as elevated levels are associated with significantly reduced patient survival. A strong relationship between HOTAIR expression and HOXA9 levels has also been documented, particularly in higher-grade tumors. Chromatin immunoprecipitation–qPCR further confirmed that HOXA9 directly binds to the HOTAIR promoter, supporting its transcriptional regulation by HOXA9 [76].

RNA-seq analyses from Chinese glioma cohorts identified HOTAIR among the most highly upregulated lncRNAs across tumor grades [77]. In contrast, a study reported reduced HOTAIR expression in U118, MG-U87, and MG-LN18 glioblastoma cells [78].

Using RNA-seq data from 152 patients with glioblastoma in TCGA, Lei et al. identified HOTAIR as a key component of a nine-lncRNA prognostic signature capable of predicting glioblastoma outcomes. Their bioinformatic analyses suggested that HOTAIR may influence multiple downstream targets involved in diverse pathways, including cell–cell communication, neurotransmitter regulation, and calcium signaling. Although experimental validation is still required to clarify these mechanisms, additional in silico studies similarly propose multifaceted tumor-promoting roles for HOTAIR in glioma [79]. These findings indicate that HOTAIR may serve as a promising prognostic biomarker candidate for glioma progression, severity, and molecular grade, either independently or as part of a biomarker panel. Moreover, the consistent association between high HOTAIR expression and poor clinical outcomes may represent its potential value as a therapeutic target, particularly in aggressive glioma subtypes [80]. Another related factor in gliomas is expression of MEG3. Reduced MEG3 expression has been consistently associated with poorer overall survival in patients with glioma [81]. Supporting these findings, MEG3 has also been proposed as a prognostic and immunotherapy-related biomarker [82]. Evidence highlights that MEG3 exerts its tumor-suppressive effects through lncRNA–miRNA–mRNA regulatory networks. MEG3 inhibits glioma cell proliferation, migration, and invasion by sponging miR-19a, thereby elevating PTEN expression [83]. Another study identified the MEG3/miR-96-5p/MTSS1 axis as a potential therapeutic target. MEG3 has also been reported to interact with miR-377/PTEN [84]. Similarly, MEG3 has also been reported to inhibit glioma progression through the miR-6088/SMARCB1 axis [85].

## 5. The lncRNA–Cisplatin Interplay in Glioma/GBM: Mechanistic Insights

Cisplatin (CDDP) is a widely used chemotherapeutic agent for numerous malignancies, yet the emergence of resistance substantially limits its clinical effectiveness and contributes to tumor relapse and poor survival. lncRNAs, which regulate chromatin dynamics, transcription, and post-transcriptional processes, have increasingly been implicated in the acquisition of CDDP resistance. Recent studies reveal that lncRNAs modulate key mechanisms underlying chemoresistance, including drug transport, detoxification, DNA repair, apoptosis, autophagy, stemness, and associated signaling pathways. This review summarizes current insights into lncRNA-mediated CDDP resistance and outlines potential lncRNA-targeted strategies to improve therapeutic outcomes [86]. The related resistance mechanisms are shown in Table 1.

### 5.1. Modulating Apoptosis and Cell Survival Pathways

Telomerase reverse transcriptase (TERT), the catalytic subunit of telomerase, is essential for telomere maintenance. MALAT1 is upregulated in glioma and promotes malignant phenotypes, while in cisplatin-resistant GBM cells it has been reported to enhance survival via PI3K/AKT activation and reduced apoptosis. FOXD2-AS1 is also highly expressed in glioma and promotes proliferation and migration while inhibiting apoptosis. Collectively, increased expression of MALAT1 and FOXD2-AS1 may support sustained cellular proliferation in glioma, although direct links to telomerase regulation and cisplatin resistance require further validation [89,90,91].

In glioma, ANRIL expression is inversely correlated with miR-203a, and ANRIL silencing affects cell-cycle progression and apoptosis-related phenotypes. SNHG3, SNHG6, SNHG16, and SNHG20 have also been reported to promote glioma progression by repressing tumor-suppressive pathways, including PTEN/PI3K/AKT- and p21-associated signaling, thereby favoring proliferation and reducing apoptosis. Collectively, these lncRNAs contribute to glioma progression through regulation of apoptosis, cell-cycle control, and oncogenic signaling pathways [92,93].

In addition, DANCR was found to be highly expressed in glioma tissues and cells, suggesting an oncogenic function. Kaplan–Meier analysis showed that elevated DANCR levels were associated with poorer overall survival. Functional assays (MTT, colony formation, and transwell) demonstrated that silencing DANCR can reduce glioma cell proliferation and migration. Western blot analysis further revealed that DANCR knockdown suppressed key proteins of the Wnt/β-catenin pathway, while activation of this pathway reversed the inhibitory effects. Overall, the findings indicate that DANCR promotes glioma progression by activating the Wnt/β-catenin signaling pathway, and also activates AXL/PI3K/Akt/NF-κB in U251/U87 cisplatin-resistant lines [25]. Another factor is HOXD-AS1, which has been identified as an oncogenic factor in various cancers, although its role in glioma and cisplatin (DDP) resistance remained unclear. This study showed that HOXD-AS1 is highly expressed in glioma tissues and cells, and its elevated levels were associated with poorer survival. Silencing HOXD-AS1 reduced glioma cell proliferation, migration, and invasion, and increased sensitivity to DDP. Mechanistically, HOXD-AS1 acts as a molecular sponge for miR-204. Overexpressing miR-204 produced similar tumor-suppressive effects as HOXD-AS1 knockdown, while miR-204 inhibition counteracted the impact of HOXD-AS1 silencing. Overall, HOXD-AS1 promotes glioma progression and DDP resistance by sequestering miR-204, highlighting its potential as a therapeutic target [94]. Regarding other lncRNAs, several lncRNAs (e.g., MEG3 and GAS5) have been reported to influence apoptosis and autophagy in glioma and modulate chemosensitivity in non-CDDP contexts.

GAS5 regulates miR-222 in glioma and suppresses tumor malignancy by downregulating miR-222, which upregulates tumor suppressors involved in apoptosis, migration, and invasion. The GAS5/miR-222/PTEN axis has been demonstrated in gastric cancer, but its role in chemosensitivity in glioma remains unproven [95]. GAS5 also forms a positive feedback loop with miR-196a-5p and FOXO1 in glioma stem cells, suppressing malignancy. While GAS5 has been shown to promote M1 polarization of TAMs in endometrial cancer via miR-21–PTEN–AKT signaling, there is no evidence that the GAS5/miR-196a-5p/FOXO1 axis regulates TAMs in glioma [96]. Emerging research methodologies can also enhance the study of GAS5. Advanced technologies, such as single-cell transcriptomics, may offer novel insights into the molecular mechanisms underlying glioma, including the functional role of GAS5. Overall, integrating both established techniques and innovative approaches will be essential for advancing our understanding of GAS5-mediated regulatory networks in glioma [95].

Maternally expressed gene 3 (MEG3) lncRNA has been implicated in multiple cancers, yet its involvement in cisplatin-induced apoptosis in glioma remains unclear. In U87 glioma cells, MEG3 expression increased following cisplatin exposure. Lentiviral overexpression of MEG3 enhanced cisplatin sensitivity, whereas MEG3 knockdown via siRNA conferred greater resistance to the drug. Mechanistic analyses showed that MEG3 suppresses cisplatin-induced autophagy, and this reduction in autophagic activity contributes to the improved chemosensitivity of U87 cells [97].

miR-205 enhances cisplatin sensitivity of glioma cells by suppressing E2F1; downregulation of miR-205 confers cisplatin resistance via E2F1 upregulation. miR-136 does not induce cisplatin resistance in glioma via a comparable E2F1 mechanism; in glioma, it generally acts as a tumor suppressor, inhibiting proliferation and inducing apoptosis by targeting AEG-1 and BCL2, and increasing temozolomide sensitivity. Conversely, miR-873 overexpression enhances apoptosis in cisplatin-resistant glioma cells and sensitizes them to cisplatin by targeting BCL2. Glioma tissues exhibit reduced miR-873 and increased BCL2 relative to the normal brain, indicating an inverse relationship, and miR-873 modulates cisplatin sensitivity via BCL2 regulation [98,99].

### 5.2. Regulating DNA Damage Repair Machinery

Alterations in XPA expression are thought to influence cancer risk, prognosis, and treatment response. Higher XPA expression is associated with improved overall survival and favorable prognosis (*p* < 0.0001). However, in glioblastoma, high XPA also confers temozolomide resistance by protecting cells from temozolomide-induced cell death and apoptosis, highlighting a context-dependent role in treatment response. In germ cell tumors, increased XPA may contribute to cisplatin resistance and serves as an independent prognostic biomarker for OS. The NER pathway repairs bulky DNA lesions induced by UV radiation and chemotherapeutic agents; low expression of NER-related genes (including ERCC1 and XPA) can reduce DNA repair capacity and increase cancer susceptibility in certain contexts. However, this does not imply uniform prognostic benefit across all tumor types: in glioma, higher XPA correlates with better survival, whereas in other cancers, elevated XPA may favor resistance to DNA-damaging therapy [100,101,102]. Demonstrations that specific lncRNAs alter NER activity to modulate GBM cisplatin sensitivity are sparse. A conservative assessment is warranted: lncRNAs can regulate expression of repair genes in other systems, but causal GBM/CDDP NER modulation by a defined lncRNA remains to be robustly shown. Focused assays (platinum adduct quantification, γ-H2AX kinetics, or comet assays combined with lncRNA perturbation in U87/U251 ± CDDP) are required to establish direct links. Until such data are available, DNA-repair-related lncRNA effects on cisplatin responsiveness in GBM should be considered putative [103]. XPA expression is dynamically regulated at both transcriptional and post-transcriptional levels, with significant clinical implications. (NER) capacity, often due to lower XPA levels, has been linked to increased sensitivity to CDDP in malignancies, and although cisplatin induces similar DNA adducts across cell types, differences in cellular sensitivity are largely attributable to variations in DNA repair capacity and damage tolerance mechanisms. Tissue-specific transcriptional and epigenetic programs may contribute to organ-specific differences in DNA repair capacity and cisplatin responsiveness [104,105].

### 5.3. Influencing Drug Influx and Efflux Transporters

**Figure 1:**
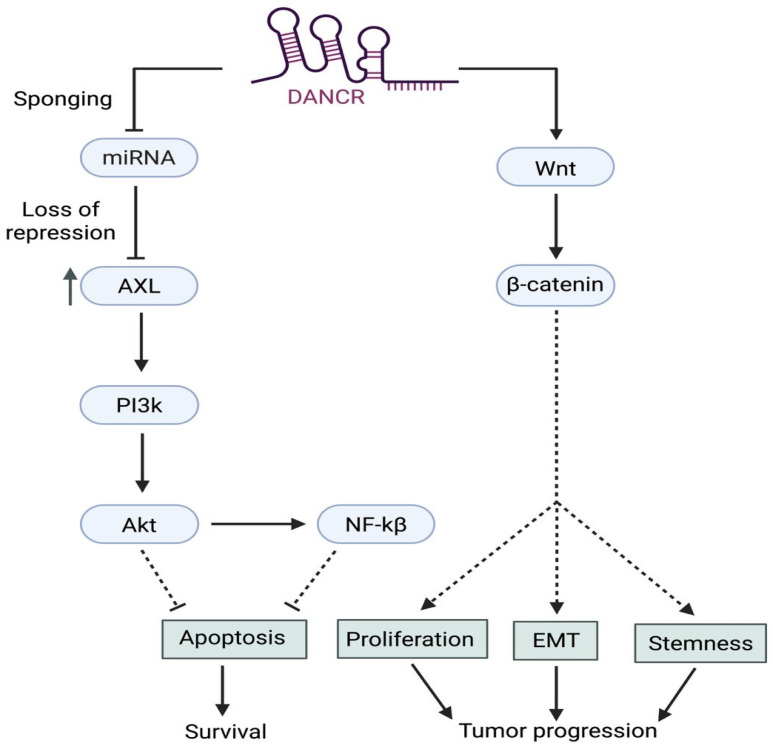
Pathways driven by DANCR that promote tumor survival and proliferation. DANCR functions as a ceRNA, sponging tumor-suppressive miRNAs, causing the loss of miRNA-mediated repression and upregulation of target genes, such as AXL. Elevated AXL activates downstream PI3K/AKT signaling, which promotes NF-κB activation, resulting in increased proliferation and inhibition of apoptosis. DANCR enhances Wnt/β-catenin signaling, leading to activation of genes associated with EMT, stemness, and proliferation.

In glioblastoma, both the structural and functional properties of the blood–brain barrier (BBB) are substantially altered. This disruption is evidenced by reduced expression of several BBB-associated proteins—including Claudin-5, GLUT1, Na^+^/K^+^-ATPase, and the major efflux transporters ABCB1 and ABCG2—within glioblastoma-associated microvessels compared with normal brain vasculature. The markedly reduced expression of Claudin-5, a key tight-junction protein, suggests impaired tight-junction integrity and increased paracellular permeability within the tumor vasculature. Likewise, decreased levels of GLUT1, an endothelial marker, and Na^+^/K^+^-ATPase, a marker of endothelial membrane specialization, likely reflect the abnormal, disorganized, and dysfunctional neovasculature characteristic of glioblastoma [106]. The substantially reduced expression of ABCB1 and ABCG2 further suggests impairment of the biochemical barrier, particularly its capacity for active efflux transport. Together with disruption of the physical BBB, these alterations may facilitate increased intratumoral accumulation of therapeutic agents that are substrates of these transporters. Consistent with this concept, the Wee1 inhibitor AZD1775 demonstrated favorable tumor penetration in patients with glioblastoma, achieving a median unbound tumor-to-plasma ratio of 3.2, despite being a known substrate of both ABCB1 and ABCG2 [107]. The modest efficacy of ABCB1 inhibitors in enhancing CNS drug permeability is largely attributed to the challenge of achieving unbound systemic inhibitor concentrations high enough to produce meaningful transporter inhibition in humans. Furthermore, it is important to recognize that ABCB1 and ABCG2 function cooperatively at the BBB, jointly restricting the brain uptake of compounds that serve as dual substrates for both transporters (Figure 1).

Another item is OATP1A2, which is often cited as the primary OATP isoform present at the human BBB. Immunofluorescence studies have localized OATP1A2 to the luminal surface of human brain endothelial cells, and both OATP1A2 and OATP2B1 have been reported in endothelial cells within human glioma tissue. However, quantitative targeted proteomic analyses from independent groups, including our own, have demonstrated that the protein levels of OATP1A2 and OATP2B1 in isolated microvessels from human brain and glioblastoma samples fall below the lower limit of quantification (<0.1 fmol/μg), suggesting minimal or undetectable expression [106].

These differential patterns of transporter expression between the healthy BBB and glioblastoma vasculature not only provide mechanistic insight into barrier dysfunction, but also contribute to quantitative frameworks for modeling heterogeneous drug delivery to the brain and glioblastoma tissue. A major mechanism of chemoresistance in many tumors is the reduced intracellular accumulation of anticancer drugs. Because ABC transporters are key mediators of drug efflux in resistant cancer cells, considerable effort has focused on developing agents capable of inhibiting or modulating their activity to enhance intracellular drug levels. Although the regulation of ABC transporters is complex and influenced by multiple molecular pathways, lncRNAs have emerged as important regulators of ABC transporter expression and function, contributing to either the development or reversal of chemoresistance [108]. Consequently, elucidating the molecular mechanisms, signaling pathways, and regulatory networks governing ABC transporter activity represents a promising strategy for improving treatment responses in patients with poor prognoses and for advancing the development of lncRNA-based anticancer therapeutics [109].

lncRNAs influence ABC transporter–mediated chemoresistance through multiple mechanisms, including regulating transcription factor binding and translocation, modifying chromatin structure, altering promoter accessibility, interacting with RNA-binding proteins, and modulating epigenetic marks [110]. Among these regulatory elements, STAT3 and FOXC2 have been reported as transcriptional regulators involved in lncRNA-associated ABCB1 regulation in specific cancer contexts [111]. The PI3K/Akt/mTOR signaling axis also contributes to lncRNA-mediated modulation of P-gp activity. For example, HOTAIR enhances PI3K, AKT, and mTOR phosphorylation while increasing P-gp protein levels, thereby reducing doxorubicin cytotoxicity that is associated with activation of PI3K/AKT/mTOR signaling and increased P-gp expression [112]. This suggests a functional connection between PI3K/Akt/mTOR signaling and P-gp expression in lncRNA-associated drug resistance, highlighting the potential for therapeutic strategies targeting both pathways. Given the epigenetic regulation of ABC transporters, numerous lncRNAs interact with epigenetic components during chemoresistance [113,114]. EZH2, the catalytic subunit of PRC2 responsible for H3K27 trimethylation and transcriptional repression, has been implicated in epigenetic regulation of chemoresistance pathways in glioma, including modulation of ABC transporter expression and temozolomide response [109].

### 5.4. Controlling Epithelial–Mesenchymal Transition (EMT) and Invasiveness

Studies using lncRNA overexpression or silencing in GBM models have demonstrated that lncRNAs influence epithelial–mesenchymal transition (EMT)-like phenotypes through involvement in Wnt signaling, either by directly interacting with pathway components or indirectly via regulators, such as ZEB transcription factors and VEGF, forming interconnected regulatory networks that contribute to sustained pathway activation. The PI3K/Akt cascade, which regulates numerous downstream effectors, also contributes to dysregulated angiogenesis and mesenchymal (MES) transition, both of which are essential for GBM progression. These pathways are major therapeutic targets in GBM [115]. Individual lncRNAs typically regulate multiple downstream molecules and frequently participate in interconnected signaling networks. For instance, the MES-associated transcription factor ZEB1 is regulated by several lncRNAs, including UCA1 and LINC00645, with emerging evidence for additional candidates, such as OR7E156P, through miRNA-mediated regulatory axes targeting upstream hypoxia-responsive elements [116]. Consistent with these regulatory networks, induction of EMT-like programs in glioma cells in vitro has been associated with increased tissue factor (TF) expression. Further analyses show that ZEB1 expression correlates positively with glioma grade and TF levels in tumor tissues. Mechanistic studies suggest that EMT-associated signaling may enhance TF expression through the miR-200a/ZEB1 axis. Collectively, these findings indicate that EMT-like transitions may promote TF expression in glioma and suggest that TF could represent a potential therapeutic target for inhibiting EMT-associated progression and thrombotic complications [117].

### 5.5. Interplay with Key Signaling Pathways

The canonical Wnt/β-catenin signaling pathway is aberrantly activated in many types of tumors [118,119]. High expression of lncRNA ST7-AS1, MIR22HG, and CASC7 has been shown to suppress the Wnt/β-catenin signaling axis by regulating levels of key proteins, thereby significantly inhibiting glioma growth [120,121,122]. Consistently, Zhou et al. reported that ADAMTS9-AS1 is associated with regulation of glioma cell proliferation and modulation of Wnt/β-catenin signaling and PCNA expression, although the precise mechanistic direction may vary depending on the experimental context [123]. Different lncRNAs exert distinct effects on the Wnt/β-catenin pathway [124]. Similarly, lncRNA NEAT1 has been shown to recruit EZH2, leading to repression of GSK3β expression and activation of the Wnt/β-catenin pathway [72]. Preliminary evidence also suggests that lncRNA CCND2-AS1 regulates glioma proliferation through modulation of Wnt/β-catenin signaling, although further mechanistic studies are required [125].

In addition to Wnt signaling, lncRNAs also regulate the PTEN/PI3K/AKT signaling axis. The focally amplified lncRNA on chromosome 1 (FAL1) has been reported to promote tumor cell proliferation, migration, and invasion through modulation of PTEN expression and activation of PI3K/AKT signaling, leading to downstream phosphorylation of GSK-3β, a key component of the Wnt pathway [126]. Studies have shown that inhibition of PI3K/AKT signaling suppresses proliferation and induces apoptosis in glioma models. For example, lncRNA MEG3 inhibits the PI3K/AKT/NF-κB signaling pathway and triggers apoptosis [127]. Overall, lncRNAs influence glioma biology through diverse mechanisms, including miRNA sponge activity, protein degradation/inactivation, modulation of RNA-binding proteins, and recruitment of chromatin-modifying enzymes, ultimately promoting or inhibiting key signaling pathways that govern tumor growth and progression [125].

### 5.6. The Role of lncRNAs in Glioma Stem-Cell Maintenance and Resistance

SOX2 plays a critical role in early embryonic development and normal tissue homeostasis by maintaining stem cell pluripotency and directing cell fate decisions. In glioma, SOX2 is essential for preserving stem-like characteristics of tumor cells and is associated with tumor aggressiveness and chemoresistance, particularly to temozolomide. Although SOX2 can promote cisplatin resistance through Wnt/β-catenin signaling in some non-glioma cancers, that mechanism is not established here for glioma. SOX2 is often positively linked with SOX9 in glioblastoma, and high SOX2 expression serves as a prognostic marker in several cancers, including glioma [128,129].

**Figure 2:**
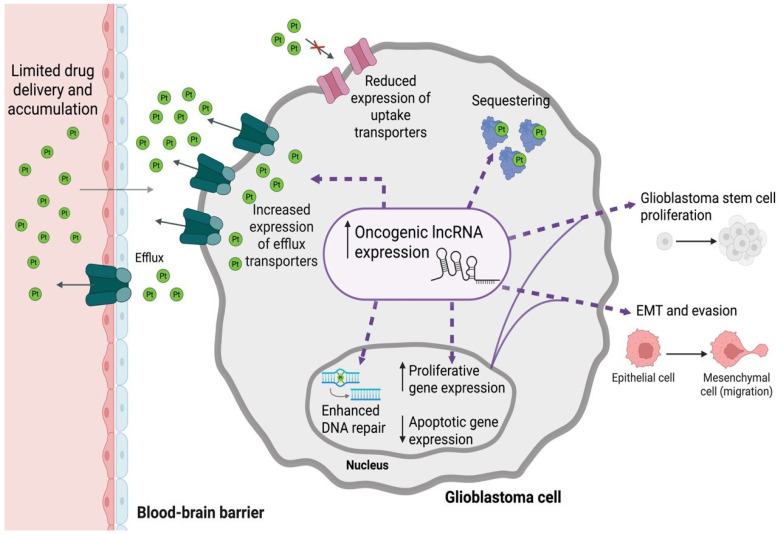
lncRNAs promote cancer progression and cisplatin (Pt) resistance in glioblastoma. Schematic overview of the mechanisms by which increased oncogenic lncRNA expression induces cisplatin resistance in glioblastoma cells. Limited drug delivery across the BBB, combined with reduced expression of uptake transporters and upregulation of efflux transporters, results in decreased intracellular cisplatin accumulation. Intracellularly, cisplatin can be sequestered, further reducing its cytotoxic effects. In the nucleus, lncRNAs promote enhanced DNA repair (e.g., nucleotide excision repair), increase expression of proliferative genes, and suppress apoptotic pathways. lncRNAs contribute to EMT-like/mesenchymal transition programs, cellular invasion, and maintenance of glioblastoma stem-like cells, cellular invasion, and the maintenance and proliferation of glioblastoma stem-like cells. Together, these processes reduce cisplatin efficacy and drive tumor survival and progression.

A study suggests that elevated SOX2OT expression promotes TMZ resistance by enhancing cell proliferation and inhibiting apoptosis. Mechanistically, SOX2OT upregulates its downstream target SOX2 via ALKBH5, an m^6^A demethylase that binds to the SOX2 promoter and increases its demethylation. Elevated SOX2 then drives proliferation and suppresses apoptosis through activation of the Wnt5a/β-catenin signaling pathway. GBM patients with higher SOX2OT expression show an increased risk of recurrence and poorer clinical outcomes compared with those with lower SOX2OT expression [128]. Additionally, H19 knockdown suppresses NF-κB signaling, as shown by reduced NF-κB reporter activity and lower expression of its downstream targets, whereas H19 overexpression activates the NF-κB pathway. Pharmacological inhibition of NF-κB significantly reverses the TMZ resistance induced by H19 overexpression. H19 expression is inducible under both short-term and long-term oxidative stress. Treatment with H_2_O_2_, which induces oxidative stress and upregulates H19, largely reduces the enhanced TMZ sensitivity caused by H19 knockdown. These findings suggest a mechanistic cascade in which oxidative stress upregulates H19, which then promotes TMZ resistance through activation of the NF-κB signaling pathway (Figure 2) [130].

### 5.7. lncRNAs as Competing Endogenous RNAs (ceRNAs) in Cisplatin Resistance

Overexpression of the PTEN 3′UTR increases the expression of 13 ceRNAs, elevates PTEN protein levels, and reduces glioma cell growth. Conversely, knockdown of any of these 13 genes decreases PTEN 3′UTR luciferase activity and significantly promotes glioma cell proliferation. The silencing effect mediated through the ceRNA mechanism is comparable to that achieved by siRNA-mediated PTEN suppression [131]. Moreover, PTEN forms a ceRNA subnetwork with several established drivers of glioma tumorigenesis and GBM subtype specification. Ectopic expression of the 3′UTRs of genes within this subnetwork results in coordinated upregulation of the other network members. Collectively, these findings indicate that the ceRNA mechanism enables concerted expression of multiple oncogenic drivers through competition for a shared miRNA pool, thereby contributing to high-grade gliomagenesis [132]. The discovery of pseudogene-mediated ceRNA networks (ceRNETs) provided the foundation for the ceRNA hypothesis [133]. However, the characterization of pseudogene-mediated ceRNETs in glioma remains limited compared with lncRNA- and circRNA-mediated networks. PTEN pseudogene-1 (PTENP1), the PTEN pseudogene, has been shown to inhibit cancer progression [134] via the ceRNA mechanism. While PTENP1 has been implicated in regulating glioma cell proliferation and invasion as a tumor suppressor, direct mechanistic evidence that its anti-tumor effects in glioma are mediated specifically through a ceRNA mechanism remains limited [135]. Additionally, a study reported the importance of the CCAT2/miR-424/Chk1 axis in shaping glioma cell behavior, so this pathway shows potential as a therapeutic target. However, several experimental limitations should be addressed. While the findings suggest that modulating the CCAT2/miR-424/Chk1 axis can improve glioma chemosensitivity, the precise regulatory mechanisms require deeper investigation [136]. In a glioblastoma xenograft mouse model, elevated LINC00479 expression was strongly associated with accelerated tumor growth. Mechanistic analyses demonstrated that LINC00479 functions as a sponge for miR-134, leading to upregulation of c-Myc, which in turn induces the multidrug resistance transporter ABCC1 and promotes temozolomide resistance [137]. A study further identified a positive co-expression pattern between MYC and ABCC1, showing that MYC overexpression can directly enhance ABCC1 expression in glioma cells [133]. DANCR functions as a competing endogenous RNA by sequestering tumor-suppressive miRNAs, thereby relieving repression of downstream oncogenic targets. In glioma, DANCR-mediated miRNA sponging has been linked to AXL/PI3K/AKT/NF-κB and Wnt/β-catenin signaling. Although PTEN-regulating lncRNA ceRNA networks may contribute to drug resistance through effects on survival, apoptosis, and DNA repair, direct evidence tying PTEN-centered ceRNA networks specifically to cisplatin resistance in glioblastoma remains limited [21,138]. Because PTEN negatively regulates PI3K/AKT signaling, disruption of PTEN-centered ceRNA networks may influence glioma cell survival and responsiveness to cisplatin, although direct experimental evidence remains limited. The lncRNA targets and mechanisms are demonstrated in Table 2.

## 6. Clinical Implications and Therapeutic Potential

### 6.1. lncRNAs as Prognostic and Predictive Biomarkers for Cisplatin Response

lncRNAs have emerged as promising candidate biomarkers, offering significant prognostic and predictive insights regarding cisplatin response in glioma and GBM. Their stable expression profiles and involvement in chemoresistance underscore their potential integration into contemporary molecular stratification frameworks. Elevated levels of oncogenic lncRNAs—such as H19, which activates the miR-29a/STAT3 signaling pathway [139], HOTAIR, which recruits PRC2 to repress DNA-damage-responsive genes [140], and MALAT1, which facilitates epithelial–mesenchymal transition and autophagy [141]—are consistently associated with reduced sensitivity to cisplatin and shorter overall survival rates. In contrast, decreased expression of tumor-suppressive lncRNAs—including MEG3, which negatively regulates the PI3K/AKT signaling pathway [84], and GAS5, which enhances apoptotic signaling—is associated with poorer therapeutic outcomes [142].

Clinically, lncRNA signatures may improve prediction of treatment response and help identify patients with glioma who are less likely to benefit from cisplatin-based regimens, although routine clinical use remains unproven. Circulating and exosomal lncRNAs, including lnc-TALC, offer promising minimally invasive platforms for monitoring evolving resistance over time [143]. From a therapeutic perspective, lncRNAs are compelling molecular targets. Strategies such as antisense oligonucleotides (ASO), RNA interference technologies, and CRISPR-based systems offer feasible means to silence oncogenic lncRNAs or restore the expression of tumor-suppressing lncRNAs [144,145,146]. These approaches can resensitize glioma cells to cisplatin treatment. Collectively, these diagnostic and therapeutic implications underscore the promise of lncRNA-directed strategies to enhance precision oncology and improve patient outcomes in GBM.

### 6.2. Therapeutic Targeting of Oncogenic lncRNAs (lncRNA Inhibition)

The therapeutic inhibition of oncogenic lncRNAs has emerged as a compelling strategy to combat cisplatin resistance in glioma and glioblastoma. This approach utilizes advanced gene-silencing modalities such as CRISPR-based editing, ASOs, and siRNAs. CRISPR/Cas9 may allow for precise genomic disruption of lncRNA loci, facilitating the permanent inactivation of chemoresistance drivers like H19, HOTAIR, and MALAT1. These lncRNAs may modulate critical pathways, including STAT3 signaling, PRC2-dependent chromatin remodeling, and autophagy [147]. Preclinical studies in multiple cancer models suggest that lncRNA inhibition may reduce stemness and restore cisplatin sensitivity, although direct GBM evidence remains limited for several targets [148]. ASOs, including gapmers, can promote RNase H–mediated degradation of specific lncRNAs, while siRNA-based approaches provide transient but potentially effective knockdown. However, delivery across the blood–brain barrier remains a major challenge, and nanoparticle or exosome-based carriers are still under active investigation. Collectively, these gene-silencing strategies represent a developing therapeutic landscape for glioma, but their translation to routine GBM therapy remains early [149,150]. Trabedersen, one of the key ASOs, was due to be evaluated to target the GBM cells as monotherapy, but the trials were not completed due to insufficient patient recruitment. However, in the previous phases, it showed a significant tumor response in patients with GBM [151]. The enhanced chemical stability and blood–brain barrier permeability of these molecules, along with their ongoing clinical applications in neurological diseases, further underscore their promise for GBM therapy. In addition, siRNA-based targeting presents a transient yet highly effective method for the suppression of lncRNAs [152]. The siRNA-mediated silencing of HULC and CRNDE has been shown to inhibit autophagy, disrupt glycolytic reprogramming, and interfere with PI3K/mTOR signaling, effectively reversing phenotypes associated with cisplatin resistance [122,153]. Innovative nanoparticle carriers—including lipid nanoparticles and exosome-mimetic vesicles—significantly enhance siRNA delivery across the blood–brain barrier and improve stability within the tumor microenvironment [154]. Collectively, these gene-silencing strategies illustrate a rapidly evolving therapeutic landscape, wherein targeted lncRNA inhibition may serve to complement standard chemotherapy, overcome resistance, and facilitate the development of personalized interventions for glioma and glioblastoma multiforme.

### 6.3. Therapeutic Restoration of Tumor-Suppressor lncRNAs

Viral vector–based delivery systems, particularly adenoviral and AAV vectors, have shown potential in restoring lncRNAs in human cells due to their high transduction efficiency and long-term expression [155]. In addition to direct gene replacement, engineered viral vectors that utilize tumor-specific promoters, capsids capable of crossing the blood–brain barrier, and microRNA response elements enhance both safety and precision in the restoration of glioma-specific long non-coding RNA (lncRNA). AAV serotypes, such as AAV2, AAV9, and AAVrh, have been explored for CNS delivery, but their efficiency depends on serotype, dose, and route of administration [156]. Lentiviral vectors can support stable expression of tumor-suppressive lncRNAs, but systemic delivery to GBM is limited by BBB constraints [128]. In parallel, non-viral approaches such as ASOs, RNA interference, and CRISPR-based systems may be used to restore tumor-suppressive lncRNAs like MEG3 and GAS5. Together, these strategies highlight the therapeutic potential of lncRNA restoration for overcoming chemoresistance and improving GBM treatment [144,145,146,157].

### 6.4. Combinatorial Strategies: lncRNA Targeting to Re-Sensitize Tumors to Cisplatin

Emerging evidence indicates that combinatorial strategies, which integrate lncRNA modulation with conventional chemotherapeutics, can effectively address cisplatin resistance in glioma and GBM. Various studies have confirmed that the combination of lncRNAs and cisplatin can have a synergistic anti-tumor effect in various human cancers. However, there is a need to explore the potential combination of lncRNAs with cisplatin to assess the sensitivity of GBM cells to cisplatin-based chemotherapy regimens.

## 7. Limitations and Challenges

Progress in this area has been fast, yet several roadblocks keep limiting the translation of lncRNA discoveries into usable therapy for GBM. At the level of basic research, the mechanistic picture remains incomplete. A handful of lncRNAs linked to cisplatin response are reasonably well-characterized [158]. These examples stand alone, though. Vast sets of lncRNA-linked systems—EMT programs, tumor stem-cell traits, and ceRNA interactions—remain only partly defined in GBM. How these circuits behave together when cells are exposed to chemotherapy remains unclear; whether any shared organization exists between tumors is also uncertain. The difficulty largely reflects the biological properties of lncRNAs. Many are weakly expressed, sharply tissue-restricted, and show little sequence conservation [68]. Thus, standard inference strategies fail, as a newly identified transcript rarely resembles anything functionally annotated. Results vary wildly across models: knocking down a single lncRNA can shift cisplatin response in one GBM line and do essentially nothing, or cause the opposite effect in another. Genetic background and even culture conditions can flip results. Reproducibility suffers, and confirming a given lncRNA as a cisplatin-resistance driver demands consistent findings across several independent systems, which remains rare.

Clinical translation faces several major barriers. One of the hardest is delivery to the brain: the BBB and blood–tumor barrier substantially limit systemic access, and even viral vectors, nanoparticles, or exosomes may fail to reach invasive tumor cells efficiently [67,68]. Therefore, ASO-, CRISPR-, and siRNA-based lncRNA therapies remain constrained by incomplete delivery to their intended targets. After delivery, safety and regulatory requirements become more stringent because nucleic-acid therapeutics can persist, trigger innate immune activation, silence unintended genes, or produce toxicity. Biomarker development is another weak point. Although many lncRNAs are altered in GBM, only a small subset has been linked specifically to cisplatin response, and many dysregulated transcripts are not sufficiently disease-specific for high-precision diagnosis [159]. As of now, there is no validated lncRNA signature that predicts cisplatin responsiveness or identifies patients likely to benefit from lncRNA-directed approaches. Without reliable markers, clinical trial design becomes inefficient. This intersects with wider problems in GBM trials: strong tumor heterogeneity, limited enrollment, and the absence of reliable early pharmacodynamic readouts complicate evaluation of experimental strategies. Weak biomarker panels together with suboptimal trial structures widen the translational gap. All these points indicate that the field is still early in development. Considerable technical progress will be needed: stronger in vivo and 3D systems (brain organoids, for example); improved delivery tools capable of crossing the BBB; deeper genomic annotation; and more adaptable trial designs. Only sustained work across these fronts will move the field forward.

## 8. Conclusions

A complex axis linking lncRNAs to cisplatin resistance is becoming clear in GBM. Many transcripts act as microRNA sponges or structural frameworks that converge on PI3K/Akt, NF-κB, and Wnt pathways to maintain survival under drug exposure. Among these, DANCR stands out. It is consistently upregulated in aggressive gliomas and associates with poorer survival [25]. Experimental evidence indicates that DANCR enforces resistance by absorbing multiple tumor-suppressive miRNAs, thereby increasing AXL expression and activating PI3K/Akt and NF-κB cascades, which block cisplatin-triggered apoptosis [21]. DANCR may also activate the Wnt/β-catenin pathway independently, promoting proliferation and survival [25]. By considering PI3K/Akt, NF-κB, and Wnt routes together, DANCR may function as a cisplatin response.

The available evidence suggests that DANCR and several other lncRNAs participate in molecular pathways associated with cisplatin resistance in glioma. However, most findings derive from in vitro studies and require validation in clinically relevant animal models and patient-derived systems. Significant challenges remain, including efficient delivery across the blood–brain barrier, tumor heterogeneity, and limited clinical evidence. Therefore, while lncRNA-targeted therapies represent a promising research direction, their clinical translation remains at an early stage.

## Figures and Tables

**Table 1:** Summarized effect of cisplatin in GBM.

Resistance Mechanisms	Key Events in GBM/Cisplatin Resistance (from This Section)	Effect on Cisplatin/Therapy	References
Limited delivery of brain tissue	BBB acts as a physical/biological barrier that reduces brain exposure to drugs; cisplatin has reduced ability to cross the BBB and the dose remains pharmacologically active due to protein binding; ABC transporters at the BBB further limit drug entry.	Low active cisplatin reaching GBM tissue contributes to intrinsic/acquired resistance and multidrug resistance (MDR); BBB/ABC transporters are potential targets to enhance delivery.	[32,33,34,35]
Drug efflux via ABC transporters	ABCB1 (P-gp/MDR1), ABCC1/2/4/5/6, and ABCG2 are implicated in cisplatin-resistant cells; ABCC2, ABCC5, and ABCC6 actively export cisplatin; ABC transporters have low substrate specificity.	Active efflux of cisplatin and other drugs by ABC transporters contributes to multidrug resistance and cross-resistance.	[36,37,87]
Detoxification & sequestration	Cisplatin binds to cysteine thiols of metallothionein, inactivating the drug; other resistance-related changes include increased clearance of protein damage, increased protein glycosylation, enhanced glycolysis, and dysregulated oxidative phosphorylation.	Detoxification and metabolic adaptation neutralize reactive platinum species and improve tolerance to cisplatin-induced damage.	[30]
Enhanced nucleotide excision repair (NER)	NER repairs helix-distorting lesions, including cisplatin-induced intrastrand crosslinks (major adduct type); high XPF and ERCC levels accelerate removal of these crosslinks.	Faster repair of lethal DNA adducts reduces cisplatin sensitivity; targeting DNA repair has been proposed as a therapeutic strategy in GBM.	[38,39,40,41,42,43]
HR proficiency/PARP inhibitor insensitivity	In IDH1/2-wild-type gliomas, PARP inhibitors show low tumor-suppressive effect; functional BRCA1/2-dependent homologous recombination (HR) is efficiently recruited to cisplatin-induced DNA damage sites, enabling accurate repair of double-strand breaks.	Functional HR repair in IDH-wild-type GBM reduces vulnerability to DNA damage and attenuates responses to platinum-based chemotherapy and PARP inhibitors.	[42,43,44]
Checkpoint & p53 pathway defects	Cisplatin-induced DNA adducts activate ATM/ATR and DDR, involving MAPK signaling and p53; p53 is frequently mutated/deleted, and the p53–ARF–MDM2 pathway is highly dysregulated in GBM.	Impaired p53-dependent checkpoints favor genomic instability and allow cells to escape apoptosis after DNA damage, contributing to resistance.	[31,46]
EMT & invasive/mesenchymal shift	EMT-inducing transcription factors include SNAIL/Slug, ZEB1/2 and TWIST family proteins; FHL proteins (e.g., FHL3) can promote EMT by stabilizing SNAIL1; EMT in GBM is linked to tumor initiation, plasticity, invasion, treatment resistance, and maintenance of cancer stem-like properties.	EMT promotes migration and invasion and is associated with treatment resistance and tumor recurrence in GBM.	[49,88]
Glioblastoma stem cells (GSCs)	GSCs are crucial for GBM development, maintenance, and recurrence and are resistant to standard therapies; resistance mechanisms include quiescence, high mitochondrial reserve, extensive DNA repair capacity, and localization in hypoxic niches.	GSCs survive therapy and repopulate the tumor, acting as a reservoir for relapse and a key target for new treatments.	[48,50,51]
Tumor microenvironment & hypoxia	TME, including interactions between GBM stem cells and their environment via extracellular vesicles, supports GBM pathogenesis and proliferation; hypoxia stabilizes HIF-1α, upregulates VEGF, shifts metabolism toward glycolysis with lactate-driven acidosis, and increases PD-L1 expression.	A hypoxic, metabolically reprogrammed, and immunosuppressive TME promotes tumor survival, angiogenesis, immune evasion, and the emergence of resistant clones.	[52,53]
Moxidative-stress adaptation	Increased clearance of protein damage, enhanced protein glycosylation, increased glycolysis, and altered oxidative phosphorylation are described as contributors to cisplatin resistance.	These metabolic adaptations improve tolerance to cisplatin-induced stress and diminish its cytotoxic effect.	[30]

**Table 2:** Summarized mechanisms for each lncRNA. For several lncRNAs, evidence derives from glioma progression studies, temozolomide resistance models, other malignancies, or bioinformatic analyses rather than direct cisplatin-response experiments.

lncRNAs	Main Targets/Mechanisms	Cisplatin and Other Chemotherapeutics	References
**DANCR**	Activates AXL → PI3K/Akt/NF-κB and Wnt/β-catenin signaling, promoting survival and blocking cisplatin-induced apoptosis in GBM cells.	↑ cisplatin IC50 by ~4–6-fold with reduced apoptosis in U87 and U251 cells; DANCR knockdown restores cisplatin sensitivity.	[21]
**HOXD-AS1**	Acts as a ceRNA for miR-204, de-repressing HMGA2 and reducing apoptosis in cisplatin-treated glioma cells.	HOXD-AS1 knockdown decreases cisplatin IC50 and increases apoptosis, thereby sensitizing glioma cells to CDDP.	[94]
**GAS5**	Evidence suggests that GAS5 may enhance chemosensitivity through regulation of apoptosis- and autophagy-related pathways.	GAS5 overexpression lowers cisplatin IC50 and enhances apoptosis; loss of GAS5 promotes cisplatin resistance.	[95,121]
**NEAT1**	Nuclear lncRNA that scaffolds EZH2 and activates Wnt/β-catenin signaling in glioma (no direct cisplatin data; evidence mainly from other tumors and general cisplatin-resistance studies).	NEAT1 upregulation is associated with reduced cisplatin sensitivity in non-GBM models; NEAT1 knockdown enhances cisplatin response—indirect evidence, to be cited cautiously.	[72,86]
**MEG3**	Tumor-suppressor lncRNA that inhibits autophagy and activates p53-dependent apoptosis in cisplatin-treated glioma cells.	Low MEG3 expression correlates with cisplatin resistance; MEG3 restoration suppresses autophagy and sensitizes U87 cells to cisplatin (lower IC50, higher apoptosis).	[97,127]
**H19**	Oncogenic lncRNA that activates STAT3 and pro-survival autophagy signaling (data in GBM mainly temozolomide, with some support for platinum-drug synergy in other settings).	High H19 contributes to multidrug resistance and likely reduces cisplatin sensitivity; H19 knockdown enhances chemosensitivity; evidence for CDDP in GBM remains indirect.	[86,130]
**CCAT2**	Sponges miR-424 → Chk1 upregulation and enhanced DNA-damage repair, supporting chemoresistance (includes cisplatin) in glioma.	CCAT2 overexpression increases resistance to cisplatin (higher IC50, reduced apoptosis); CCAT2 knockdown restores cisplatin chemosensitivity.	[133,136]
**LINC00470**	Acts as a ceRNA for miR-134, resulting in upregulation of c-Myc and the efflux transporter ABCC1, thereby enhancing drug efflux and promoting chemoresistance in glioma (shown mainly with temozolomide).	LINC00470 overexpression increases temozolomide IC_50_ and reduces apoptosis, whereas its knockdown restores chemosensitivity; although demonstrated primarily for temozolomide, this efflux-based mechanism is likely relevant to cisplatin response.	[133,137]

## Data Availability

No new data were created or analyzed in this study. Data sharing is not applicable to this article.

## Abbreviations

The following abbreviations are used in this manuscript:

ABC	ATP-binding cassette
ABCB1 (P-gp/MDR1)	ATP-binding cassette subfamily B member 1 (P-glycoprotein/multidrug resistance protein 1)
ABCC	ATP-binding cassette subfamily C
ABCG2 (BCRP)	ATP-binding cassette subfamily G member 2 (breast cancer resistance protein)
ALL	Acute lymphoid leukemia
ANRIL	Antisense noncoding RNA in the INK4 locus
ATM	Ataxia telangiectasia mutated
ATR	ATM and Rad3-related
ATRX	Alpha thalassemia/mental retardation syndrome X-linked
BBB	Blood–brain barrier
BCL2	B-cell lymphoma 2
BRCA1/2	Breast cancer susceptibility genes 1 and 2
CDDP	Cisplatin
ceRNA	Competing endogenous RNA
Chk1	Checkpoint kinase 1
CNS	Central nervous system
CT	Computed tomography
CTR1	Copper transporter 1
DDR	DNA damage response
EGFR	Epidermal growth factor receptor
EMT	Epithelial–mesenchymal transition
ERK1/2	Extracellular signal-regulated kinases 1/2
FOXO1	Forkhead box O1
GBM	Glioblastoma
GSCs	Glioblastoma stem cells
GSH	Glutathione
HIF-1α	Hypoxia-inducible factor 1-alpha
HR	Homologous recombination
IC50	Half-maximal inhibitory concentration
JNK	c-Jun N-terminal kinase
lncRNA	Long non-coding RNA
LOH	Loss of heterozygosity
MAPK	Mitogen-activated protein kinase
MDR	Multidrug resistance
MDM2	Mouse double minute 2
miRNA (miR)	MicroRNA
mRNA	Messenger RNA
MRI	Magnetic resonance imaging
MTT	Methylthiazolyldiphenyl-tetrazolium bromide assay
MYC (c-Myc)	MYC proto-oncogene
NER	Nucleotide excision repair
nt	Nucleotide
PARP	Poly(ADP-ribose) polymerase
PCR	Polymerase chain reaction
PDGFA	Platelet-derived growth factor A
PDGFRα	Platelet-derived growth factor receptor alpha
PD-L1	Programmed death-ligand 1
PET	Positron emission tomography
PI3K	Phosphoinositide 3-kinase
PTEN	Phosphatase and tensin homolog
qPCR	Quantitative polymerase chain reaction
RB	Retinoblastoma
RNA	Ribonucleic acid
RT	Radiotherapy
siRNA	Small interfering RNA
SPECT	Single-photon emission computed tomography
SOX2	SRY-box transcription factor 2
TERT	Telomerase reverse transcriptase
TME	Tumor microenvironment
TMZ	Temozolomide
TP53	Tumor protein p53
VEGF	Vascular endothelial growth factor
XPA	Xeroderma pigmentosum group
